# Insights on Microsatellite Characteristics, Evolution, and Function From the Social Amoeba *Dictyostelium discoideum*

**DOI:** 10.3389/fnins.2022.886837

**Published:** 2022-06-13

**Authors:** Felicia N. Williams, K. Matthew Scaglione

**Affiliations:** ^1^Department of Molecular Genetics and Microbiology, Duke University, Durham, NC, United States; ^2^Department of Neurology, Duke University, Durham, NC, United States; ^3^Duke Center for Neurodegeneration and Neurotherapeutics, Duke University, Durham, NC, United States

**Keywords:** trinucleotide repeat, neurodegenerative diseases, polyglutamine, microsatellite, *Dictyostelium*

## Abstract

Microsatellites are repetitive sequences commonly found in the genomes of higher organisms. These repetitive sequences are prone to expansion or contraction, and when microsatellite expansion occurs in the regulatory or coding regions of genes this can result in a number of diseases including many neurodegenerative diseases. Unlike in humans and other organisms, the social amoeba *Dictyostelium discoideum* contains an unusually high number of microsatellites. Intriguingly, many of these microsatellites fall within the coding region of genes, resulting in nearly 10,000 homopolymeric repeat proteins within the *Dictyostelium* proteome. Surprisingly, among the most common of these repeats are polyglutamine repeats, a type of repeat that causes a class of nine neurodegenerative diseases in humans. In this minireview, we summarize what is currently known about homopolymeric repeats and microsatellites in *Dictyostelium discoideum* and discuss the potential utility of *Dictyostelium* for identifying novel mechanisms that utilize and regulate regions of repetitive DNA.

## Introduction

Microsatellites are a universal feature of most organismal genomes, though the prevalence and characteristics of these vary widely between species. These genetic features, sometimes referred to as simple sequence repeats (SSRs), are short tandem repeats composed of 1–6 bp sequences (Ellegren, 2004). SSRs tend to be highly polymorphic and are primarily located within non-coding portions of the genome (Ellegren, 2004). Despite their ubiquity, expansion of microsatellites are known to cause several different diseases. These disease-causing expansions occur in both coding and non-coding regions of the genome, reflecting the wide array of mechanisms by which these microsatellites disrupt normal cellular functions (Ranum and Day, 2002; Orr and Zoghbi, 2007; Brouwer et al., 2009).

The social amoeba *Dictyostelium discoideum* raises new questions about the function and impact of microsatellites. These questions are raised because the *Dictyostelium* genome has a massive amount of these features with 11% of its genome composed of SSRs, about a 50-fold enrichment over most other organisms (Eichinger et al., 2005). Interestingly, unlike other organisms that encode mostly dinucleotide repeats, *Dictyostelium* encodes mostly trinucleotide repeats (Eichinger et al., 2005). The number of tandem repeats of trinucleotides (and hexa-, nona-, etc.) is also extremely high within coding regions resulting in the production of nearly 10,000 proteins that encode SSRs (Eichinger et al., 2005). Surprisingly, unlike in humans, microsatellite expansion within exons does not appear to be detrimental to *Dictyostelium* (Malinovska et al., 2015; Santarriaga et al., 2015). This raises several questions. How does *Dictyostelium* maintain genome stability? What are the functional aspects of SSRs? How is protein quality control maintained? Here, we will summarize the current knowledge of SSRs in *Dictyostelium* and describe the potential for utilizing this unique organism to explore questions in microsatellite biology.

## Microsatellite Mutation in *Dictyostelium*

The expansion and contraction of microsatellites is known to be influenced by both the composition and length of the repetitive sequence, as well as the DNA repair landscape of the cell (Schlötterer and Tautz, 1992; Strand et al., 1993; Sia et al., 1997; Lai and Sun, 2003; Shinde et al., 2003; Tian et al., 2011; Hamilton et al., 2017). Current models of microsatellite mutation attribute changes in microsatellite length primarily to slippage mutations, a phenomenon in which a newly synthesized DNA strand briefly dissociates during DNA replication but is misaligned after reannealing due to the repetitiveness of the template, resulting in some number of repeats remaining unannealed (Schlötterer and Tautz, 1992; Strand et al., 1993; Sia et al., 1997). This can result in either expansion or contraction of the microsatellite depending on which strand contains the unannealed portion of DNA (Schlötterer and Tautz, 1992; Strand et al., 1993; Sia et al., 1997). It is known that the frequency of slippage mutations occurring is dependent on the length of the repeat unit, the number of repeat units present, and the nucleotide composition of the microsatellite (Sia et al., 1997; Lai and Sun, 2003; Shinde et al., 2003). Also important in slippage mutation is the presence or absence of functional DNA repair, particularly in the mismatch repair pathway, though some have hypothesized that errors in double strand break repair by homologous recombination may also result in changes in microsatellite length (Sia et al., 1997; Richard and Pâques, 2000).

As mentioned previously, the genome of *Dictyostelium* is highly repetitive with over 11% of its genome being composed of SSRs (Eichinger et al., 2005). The genome is over 75% A + T rich, a value comparable to some other protozoa such as *Plasmodium falciparum* but far exceeding most other eukaryotes (Eichinger et al., 2005). Some have proposed that this bias is the reason for the notable prevalence of microsatellites in coding regions because it easier for point mutations to result in a codon identical to neighboring codons, thus increasing the likelihood that a region will become prone to slippage mutations (Tian et al., 2011; Scala et al., 2012). Consistent with this, a high rate of 3n indels present in regions without simple sequence repeats were found to occur in *Dictyostelium*, presumably occurring *via* slipped strand mispairings (Kucukyildirim et al., 2020). In addition, it was also observed that nearly one-third of indel events occurred in SSRs, primarily in homopolymeric A:T runs (Kucukyildirim et al., 2020). Together these provide one potential explanation for the high number of trinucleotide repeats in *Dictyostelium* with small repeats potentially being preferentially expanded, resulting in an abundance of SSRs.

Surprisingly, despite having such unusually abundant microsatellites, early studies estimated that *Dictyostelium* microsatellites tend to accumulate mutations less rapidly than most other eukaryotes (McConnell et al., 2007; Saxer et al., 2012). By these estimates, the low mutation rate would suggest that rapid mutation is not the source of these extensive microsatellites in *Dictyostelium*, though another possible explanation for the low mutation rates is that expansion and contraction of microsatellites is balanced, thus masking the effects of mutations over several generations (Saxer et al., 2012; Kucukyildirim et al., 2020). In contrast to the early studies, a later study by Kucukyildirim et al. (2020) estimated an indel mutation rate higher than most organisms and attributed this to the high A + T content of the genome. However, in *Plasmodium falciparum*, a protist with even higher A + T content and lower percentage of the genome composed by SSRs, the indel mutation rate is estimated to be many fold higher than that of *Dictyostelium* (Hamilton et al., 2017; Kucukyildirim et al., 2020). It is evident from these conflicting findings that more research is needed to uncover the mutational dynamics of SSRs in *Dictyostelium.*

It is possible that *Dictyostelium* has evolved highly efficient DNA repair pathways to prevent additional mutations (McConnell et al., 2007; Saxer et al., 2012). Being a soil-dwelling microbe means that *Dictyostelium* cells come into contact with numerous mutagenic compounds that would select for rigorous DNA repair mechanisms (Deering, 1994). *Dictyostelium* also require efficient DNA repair mechanisms due to the fact that they are professional phagocytes, a process that exposes the cells to constant challenges from the bacteria consumed (Deering, 1968; Hsu et al., 2006; Zhang et al., 2009; Pontel et al., 2016). Importantly, *Dictyostelium* shows evidence of conservation of multiple eukaryotic DNA repair pathways, including some which were once thought to be limited to vertebrate animals (Table 1; Hsu et al., 2006; Pears and Lakin, 2014; Pears et al., 2021). Though much of the research on DNA repair in *Dictyostelium* has been focused on the processes of homologous recombination and non-homologous end joining (Katz and Ratner, 1988; Hsu et al., 2006, 2011), *Dictyostelium* also contains several orthologs of genes known to be associated with mismatch repair. However, these have not been extensively studied in *Dictyostelium*. Given what we know of the relevance of these pathways in microsatellite mutation in other organisms, it is important to consider that there may be insights to be had from studying these processes in an organism such as *Dictyostelium* that demonstrates remarkably lower microsatellite mutation rates than would be expected of a highly repetitive genome.

**TABLE 1:** Orthologs of human and *S. cerevisiae* DNA repair genes in *Dictyostelium*.

Gene name	*Dictyostelium* gene ID	Human gene ID	Yeast gene ID

Homologous recombination			
*blm*	DDB_G0292130	HGNC:1058	YMR190C
*exo1*	DDB_G0291570	HGNC:3511	YDR263C
*nse1*	DDB_G0279231	HGNC:29897	YLR007W
*rad51*	DDB_G0273139 DDB_G0273611	HGNC:9817	YER095W
*rad52*	DDB_G0269406	HGNC:9824	YML032C
*smc5*	DDB_G0290919	HGNC:20465	YOL034W
*smc6*	DDB_G0288993	HGNC:20466	YLR383W
*wrn*	DDB_G0268512	HGNC:12791	YMR190C
*xpf*	DDB_G0284419	HGNC:3436	YPL022W
*xrcc2*	DDB_G0290297	HGNC:12829	–

**Non-homologous end joining**			

*adprt1A (PARP1)*	DDB_G0278741	HGNC:270	–
*adprt2 (PARP2)*	DDB_G0292820	HGNC:272	–
*dclre1(Artemis-related)*	DDB_G0277755	HGNC:17660	YMR137C
*dnapkcs*	DDB_G0281167	HGNC:9413	–
*ku70*	DDB_G0286069	HGNC:4055	YMR284W
*ku80*	DDB_G0286303	HGNC:12833	YMR106C
*lig4*	DDB_G0292760	HGNC:6601	YOR005C
*mre11*	DDB_G0293546	HGNC:7230	YMR224C
*pnkp*	DDB_G0281229	HGNC:9154	YMR156C
*rad50*	DDB_G0292786	HGNC:9816	YNL250W
*xrcc4*	DDB_G0278203	HGNC:12831	–

**Mismatch repair**			

*msh1*	DDB_G0275999	–	YHR120W
*msh2*	DDB_G0275809	HGNC:7325	YOL090W
*msh3*	DDB_G0281683	HGNC:7326	YCR092C
*msh4*	DDB_G0283957	HGNC:7327	YFL003C
*msh5*	DDB_G0284747	HGNC:7328	YDL154W
*msh6*	DDB_G0268614	HGNC:7329	YDR097C
*mlh1*	DDB_G0287393	HGNC:7127	YMR167W
*mlh3*	DDB_G0283883	HGNC:7128	YPL164C
*pcna*	DDB_G0287607	HGNC:8729	YBR088C
*pms1*	DDB_G0283981	HGNC:9122	YNL082W
*rfc1*	DDB_G0285961	HGNC:9969	YOR217W

## Do Simple Sequence Repeats Serve a Function in *Dictyostelium*?

In recent years, more and more research has been conducted to study the functional aspects of homopolymeric amino acid sequences, low-complexity domains, and prion-like domains within proteins (Alberti, 2017; Alberti et al., 2019; Franzmann and Alberti, 2019b; Lau et al., 2020; Guo et al., 2021). While some studies have found evidence for beneficial impacts of having these repetitive domains, research has not yet been conducted to assess the function of any of these features in *Dictyostelium*. Instead, the research has been focused on looking for evidence of selection acting on these domains through genomic level analysis of SSR distribution and mutational patterns (Eichinger et al., 2005; Saxer et al., 2012; Scala et al., 2012; Kucukyildirim et al., 2020). If SSRs serve a function in *Dictyostelium*, we would expect to see evidence of selection acting upon them. However, the analyses that have been performed and the conclusions they have drawn have left this question unanswered. There are many arguments for and against the presence of selection acting on SSRs.

One characteristic that favors the idea that selection is in effect is that SSRs within coding regions are often read in frames that disproportionately favor one amino acid. For example, proteins are more likely to homopolymeric runs of asparagine or glutamine than the amino acids that would be produced in the other two reading frames. Furthermore, mutations within these SSRs are often synonymous, indicating that a particular amino acid is favored over alternatives (Eichinger et al., 2005). Polyasparagine and polyglutamine tracts are overrepresented in regulatory factors such as kinases, transcription factors, and RNA binding proteins, indicating that these repetitive regions may play some sort of regulatory role within the cell (Eichinger et al., 2005). *Dictyostelium* also has a low mutation rate when compared to organisms with similar genome composition, indicating that there may be selection acting to counter the effects of genetic drift in this organism (Kucukyildirim et al., 2020).

In contrast, there is high variation and genetic diversity among amino acid repeats in coding sequences, which is unexpected in protein sequences under purifying selection. Additionally, SSRs in coding regions are equal as variable as SSRs in non-coding regions, indicating that there is not stronger selection occurring as would be expected for a functional protein sequence (Scala et al., 2012). The four amino acids most commonly found in homopolymeric tracts (asparagine, glutamine, threonine, and serine) are all polar and hydrophilic, indicating that they may be more likely to reside on the outer parts of a protein vs. the hydrophobic core (Eichinger et al., 2005; Scala et al., 2012). Low mutation rates may have evolved as a mechanism to protect cells from deleterious expansions or contractions within the genome rather than as a mechanism to preserve function in coding SSRs (Kucukyildirim et al., 2020). It is clear that additional study is required to draw a more definite conclusion on whether selection is acting upon SSRs in *Dictyostelium*. Additionally, it would be helpful to conduct directly targeted studies on the results of removing the SSRs within some of the proteins they are found in and assessing whether there are effects on fitness.

## What Can We Learn From *Dictyostelium* Microsatellites?

There are several human diseases associated with microsatellite expansion (Ranum and Day, 2002; Orr and Zoghbi, 2007; Brouwer et al., 2009). However, despite the many orthologs of human disease-associated genes and the seeming lack of harmful effects from its highly repetitive genome, relatively little research has been done in *Dictyostelium* on diseases caused by microsatellite expansion (Myre et al., 2011; Wang et al., 2011; Myre, 2012; Olmos et al., 2020; Haver and Scaglione, 2021). One microsatellite-associated disease that has been modeled in *Dictyostelium* is Huntington’s Disease. In this disease, expansion of a CAG repeat encodes a homopolymeric polyglutamine tract in the huntingtin protein (HTT) that exceeds beyond a pathogenic threshold and is prone to aggregation (Orr and Zoghbi, 2007). An ortholog of HTT exists in *Dictyostelium*, and deletion of this protein results in several abnormal phenotypes including deficiencies in chemotaxis, flaws in cytokinesis, and improper cell patterning during multicellular development (Myre et al., 2011; Wang et al., 2011; Bhadoriya et al., 2019). *Dictyostelium* HTT lacks the polyglutamine tract present in exon 1 of human HTT, instead containing a polyglutamine tract further downstream (Myre et al., 2011). Because of this *Dictyostelium* may serve as an interesting organism to use in studying the effects of the presence and absence of polyglutamine tracts in the HTT protein. *Dictyostelium* may also serve as an ideal model to assess the impacts of polyglutamine tract length on protein function, a topic of interest in recent studies (Iennaco et al., 2022).

Furthermore, if there are unknown factors in *Dictyostelium* that mitigate the deleterious effects of expanded microsatellites, we could gain novel insights on how to alleviate the impact of these in human cells. The proteome of *Dictyostelium* is rich in proteins with prion-like domains, including homopolymeric polyglutamine and polyasparagine tracts as well as low complexity domains consisting of alternating amino acid residues (Eichinger et al., 2005; Malinovska and Alberti, 2015). However, *Dictyostelium* has been shown to be resistant to polyglutamine aggregation (Malinovska et al., 2015; Santarriaga et al., 2015). Similarly, *Dictyostelium* has not been found to suffer deleterious effects from its many polyasparagine-rich or low complexity domains, though these are common features in prion proteins (Liebman and Chernoff, 2012; Franzmann and Alberti, 2019a). This begs the question of how *Dictyostelium* cells are able to tolerate these usually unstable proteins while other organisms would face protein aggregation and cytotoxicity. Has *Dictyostelium* evolved novel protein quality control mechanisms to maintain these proteins in a soluble, folded state? While this is largely an unanswered question, some evidence exists for novel mechanisms that suppress polyglutamine aggregation (Santarriaga et al., 2018). Potentially there are other mechanisms also involved in mitigating deleterious effects of these genes such as alternative splicing or gene silencing. Further research is certainly needed to clarify the mechanisms of maintaining protein homeostasis within this organism.

**FIGURE 1:**
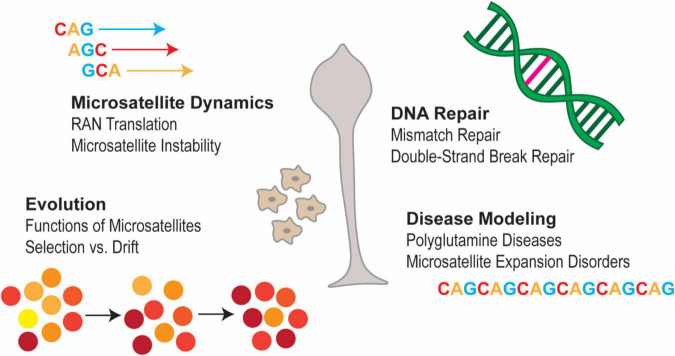
Areas to study SSRs in *Dictyostelium*. *Dictyostelium* has several unique properties that make it a compelling model in which to study the biology of SSRs. These properties include, but are not limited to, its highly repetitive genome, its genetic tractability, its high degree of genetic conservation with humans and its apparent resistance to the toxic effects of microsatellite expansion. Thus, *Dictyostelium* presents a unique opportunity to gain insights into the processes that regulate microsatellites and the biological consequences of having these repetitive sequences in the genome.

Furthermore, due to its repeat-rich genome *Dictyostelium* is an interesting organism to investigate cellular phenomena associated with expanded microsatellites in a tractable and easy-to-use organism (Figure 1; Bozzaro, 2013; Pears and Lakin, 2014; Malinovska and Alberti, 2015; Haver and Scaglione, 2021; Pears and Gross, 2021; Pears et al., 2021). Here we can begin to address many questions of relevance to human health. For instance, is there evidence of Repeat Associated Non-ATG (RAN) translation occurring in *Dictyostelium*? RAN translation is a phenomenon in which transcripts containing certain SSRs can initiate translation without the presence of an AUG start codon (Zu et al., 2011; Cleary and Ranum, 2014). These transcripts can be translated in multiple frames, leading to the production of proteins which vary in length and composition. This process has been implicated in a number of microsatellite-expansion diseases (Zu et al., 2011; Cleary and Ranum, 2014). RAN translation has not yet been studied in *Dictyostelium*, though given its highly repetitive genome and its experimental tractability, this organism would be an interesting candidate for studying this phenomenon *in vivo* and may provide unique insight into physiological functions of RAN translation. Additionally, because *Dictyostelium* is resistant to the deleterious effects of microsatellite expansion (Malinovska et al., 2015; Santarriaga et al., 2015), it provides a unique platform for studying the cellular dynamics of SSRs without cytotoxicity.

Another set of processes that would be advantageous to study in *Dictyostelium* are the various DNA repair pathways responsible for maintaining the integrity of the genome. As mentioned previously, *Dictyostelium* contains several orthologs to human DNA repair genes (Table 1), including some that are absent in *Saccharomyces cerevisiae* and other model organisms (Hsu et al., 2006; Pears and Lakin, 2014; Pears et al., 2021). Defects in DNA repair, particularly in the mismatch repair pathway, have been implicated in microsatellite mutations in several classes of disease. These include but are not limited to neurodegenerative diseases, in which microsatellites can become expanded and encode aggregation-prone pathogenic proteins, and various cancers, in which microsatellite instability can contribute to hypermutability within malignant growths (Loeb, 1994; Boyer et al., 1995; Karran, 1996; Thomas et al., 1996; Dietmaier et al., 1997; Shah et al., 2010; Jeppesen et al., 2011; Yamamoto and Imai, 2015; Schmidt and Pearson, 2016; Cortes-Ciriano et al., 2017; Baretti and Le, 2018; Maiuri et al., 2019). In cancer, defects in mismatch repair are especially important predictors of efficacy for certain chemotherapeutics and may require special therapies to address (Martin et al., 2010; Li and Martin, 2016). The *Dictyostelium* genome contains orthologs of several human genes known to be involved in mismatch repair, as well as other DNA repair pathways. However, little to no research has been done on mismatch repair in this organism. *Dictyostelium* would be a good model for studying these highly conserved processes in a simple and genetically tractable model. In doing so, we could gain vital insights on the genetic and biochemical factors that play a role in eukaryotic mismatch repair, allowing us a better understanding of the mechanisms driving human diseases such as hypermutability in cancer cells and microsatellite expansion in neurodegenerative disorders. There is even potential for discovery of novel DNA repair mechanisms that have evolved in *Dictyostelium* or have remained undiscovered in higher eukaryotes.

## Conclusion

The social amoeba *Dictyostelium discoideum* is unique among eukaryotic model organisms in that it features a highly repetitive genome without being known to demonstrate the deleterious impacts of expanded SSRs. However, several important aspects of microsatellite biology, including instability, behavior, and function have not been widely studied in this organism. Understanding biological processes in organisms with unique biological attributes can provide insights that provide novel insight into how nature has dealt with issues that cause disease in humans. Therefore, utilizing the unique benefits of model organisms such as *Dictyostelium* is important for expanding our knowledge of the processes driving cellular function.

## Author Contributions

FW wrote the initial draft of the manuscript. KS and FW revised and edited the manuscript. Both authors reviewed and approved the submitted manuscript.

## Conflict of Interest

The authors declare that the research was conducted in the absence of any commercial or financial relationships that could be construed as a potential conflict of interest.

## Publisher’s Note

All claims expressed in this article are solely those of the authors and do not necessarily represent those of their affiliated organizations, or those of the publisher, the editors and the reviewers. Any product that may be evaluated in this article, or claim that may be made by its manufacturer, is not guaranteed or endorsed by the publisher.
